# Supplementary material for: Group testing as a strategy for COVID-19 epidemiological monitoring and community surveillance
Source: PLoS Comput Biol. 2021 Mar 4;17(3):e1008726. doi: 10.1371/journal.pcbi.1008726 (PMC7932094; doi:10.1371/journal.pcbi.1008726)
Supplement: S1 Text — (PDF) [file pcbi.1008726.s001.pdf]

Supplementary Information

Vincent Brault, Bastien Mallein, Jean-Francois Rupprecht

I Fit of viral load distributions

I.1 Censored Gaussians

In this section, we present the simple mixing models of Sec I.2, as well as some complementary graphs and the estimations obtained for the parameters of this models.

I.1.1 Theorem I.1

Theorem I.1. *The estimators* $(\widehat{\mu}, \widehat{\sigma}, \widehat{q})$ *of* (μ, σ, q) *obtained by maximisation of the likelihood ratio are strongly consistent and asymptotically normal.*

The properties of the maximum likelihood estimators is a consequence of the fact that the (partially) censored Gaussian model belongs to the family of exponential laws (c.f. [1, Chapter 9] and Section I.1.1 of S1 Text). To check the quality of the approximation of the estimators by nlm, we simulate variable sizes of samples distributed according to the censored Gaussian model. The values of these estimations are plotted in Section I.1.2 in S1 Text.

Proof. To prove the lemma I.1, we observe that for every $x \in \mathbb{R}$ we have the following decomposition of the density f_X if $q > 0$:

$$f_X(x) = b(\eta) \exp\left[\langle \eta, T(x) \rangle\right], \qquad \text{(A)}$$

with $< \cdot, \cdot >$ is the scalar product, $\eta = \left(\frac{\mu}{\sigma^2}, \quad -\frac{1}{2\sigma^2}, \quad \ln q\right)$ the natural parameters,

$$T(x) = \left(x, \quad x^2, \quad \mathbb{1}_{\{x > d_{\text{cens}}\}}\right),$$

the sufficient statistics and

$$b(\eta) = \frac{1}{q + (1-q)F_{\mu,\sigma}(s)} \times \frac{1}{\sqrt{2\pi\sigma^2}} \exp\left(-\frac{\mu^2}{2\sigma^2}\right).$$

□

For the totally censured model, we have the same decomposition with the third parameters and taking $q = 0$. Thanks the decomposition in Eq (A), the (partially) censured model belongs to the family of exponential laws and the maximum likelihood estimators are strongly consistent and asymptotically normal.

I.1.2 Simulations

To study the quality of the estimators defined in Sec I.2.2, we simulated 10^4 samples of size $n \in \{10^2, 10^3, 10^4, 10^5\}$ of variables following the model $\mathcal{CN}_{d_{\text{cens}}}(0, 1, q)$ with $d_{\text{cens}} \in \{-2, -1, 0, 1, 2, 3\}$ and $q \in \{0, 0.1, 0.5, 0.9\}$. We provide boxplots estimations of the parameters in Fig A and a zoom on significant part in Fig B. Note that these parameters ($\mu = 0, |d_{\text{cens}}| \leq 3$) are very different from the ones expected for C_t values, but the model can be straightforwardly adapted by an affine transformation to measured parameters of interest.

Observe from Fig A that the estimations are generally close to the parameters but we can sometimes have very large deviations. We find that the more n increases, the better the estimator. The threshold seems to have a weak influence on the estimation of the partially censored model but, for the fully censored model, we see that the more d_{cens} increases and the more the quality of the estimators increases; especially when $d_{\text{cens}} = -2$ which represents approximately the 2.3% quantile. Note that we observe large deviations in the partially censored model when d_{cens} is equal to 2; this may seem counter-intuitive since we have access to around 97.7% of uncensored Gaussian information. However, this leaves few observations for the estimation of p (which we observe on the graphs of the last line) and this weakens in this case the model because censorship no longer really has any reason to be. We therefore recommend using the model only when the number of observations after censorship is sufficient to estimate the parameter p.

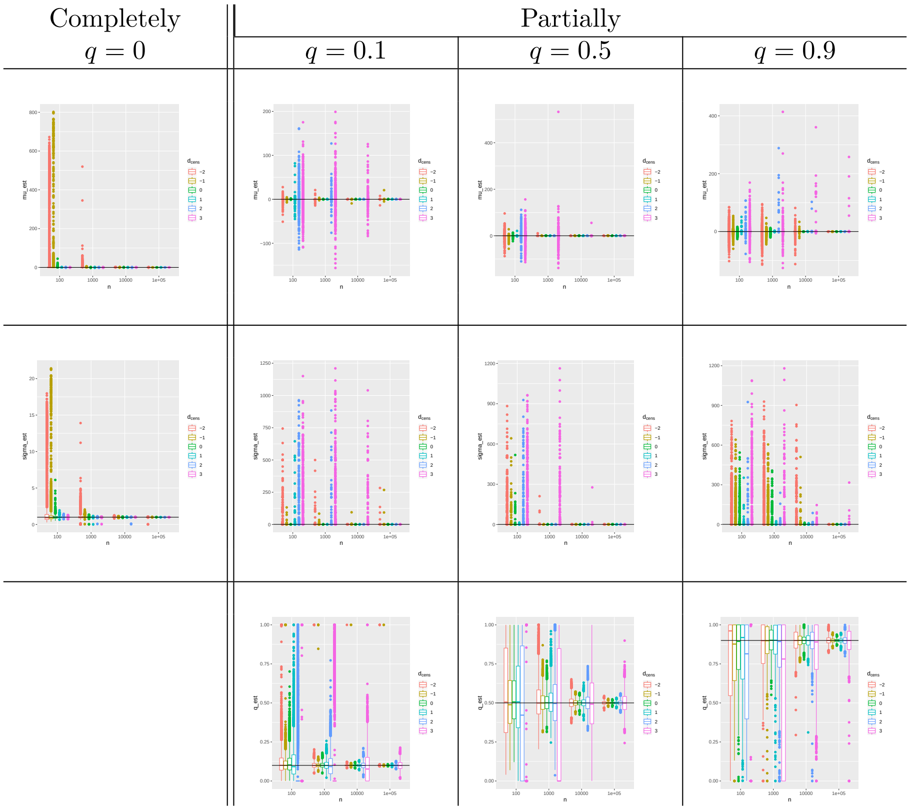

Fig A. Boxplots of the estimations of μ (first row), σ (second row) and p (last row ; only for partially censored model) in function of model (columns), the size n of sample (x-axis) and the value of the threshold s (color). The true value is symbolised by the horizontal black line. Analysis is performed on a controlled dataset.

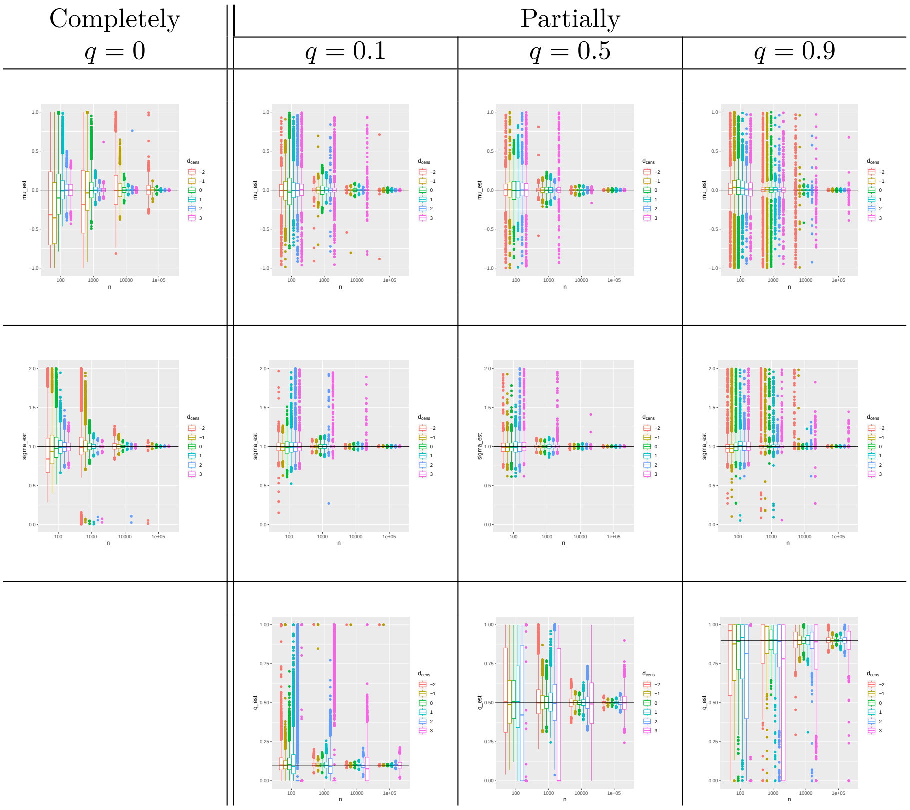

Fig B. Zoom on boxplots of the estimations of μ (first row), σ (second row) and q (last row ; only for partially censored model) in function of model (columns), the size n of sample (x-axis) and the value of the threshold s (color). The true value is symbolised by the horizontal black line. Analysis is performed on a controlled dataset.

I.2 Analysis of the Jones et al. dataset [2]

I.2.1 Naive method

In this section, we trace the density estimated by a simple mixture of Gaussian variable presented in the main text, Sec I.2.2. An estimation of the parameters of this mixture are given in Table A.

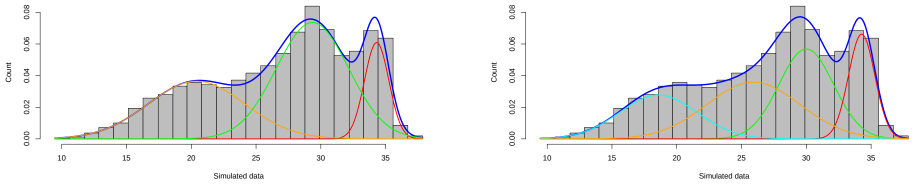

Fig C. Representation of the histogram from Jones et al. [2] with the densities estimated with 3 classes (on the left) and 4 classes (on the right): the color lines (other than blue) represent the density of each component and the blue line the density of the mixture.

As we do not have access to raw data, we performed simulations to generate a reconstructed datasets with consistent histograms to Fig 1 from [2], with randomized position of the points within each class. We applied the above procedure to 100 independently reconstructed data, in order to limit the influence of the random part. Among these 100 simulations, we obtain 95 times 3 clusters and 5 times 4 clusters. When there are 3 clusters, the estimation of the parameters is very stable (standard deviation less than 0.03 for each) but there is a little more variability in the case of 4 clusters in particular for the two classes with the largest averages (but the standard deviation does not exceed 0.25).

I.2.2 Censored mixture model

In this section, we present the complementary graphs of Sec I.2. The statistical model presented here has the following density defined for all $x \in \mathbb{R}$ by:

$$f(x) = \sum_{k=1}^{3} \pi_k \frac{f_{\mu_k,\sigma_k}(x)}{q_k + (1-q_k)F_{\mu_k,\sigma_k}(d_{\text{cens}})} \left[1 + (q_k - 1)\mathbb{1}_{\{x > d_{\text{cens}}\}}\right]. \tag{B}$$

where $f_{\mu_k,\sigma_k}(x)$ is the Gaussian density of mean μ_k and variable σ_k and $F_{\mu_k,\sigma_k}(d_{\text{cens}})$ the corresponding cumulative distribution at the limit of detection. With the model in Eq (B), we can estimate the theoretical false negative rate by the following formula:

$$\mathbb{P}\,(\text{false negative}) = \sum_{k=1}^{3} \pi_k \left[1 - F_{\mu_k,\sigma_k}(d_{\text{cens}})\right](1 - q_k). \tag{C}$$

We point out that the completely censured mixture model has the same density than the Eq (B) in the limit $q_k = 0$.

I.3 Analysis of the Lennon et al. dataset [4]

In these datasets, there are two populations : symptomatic and asymptomatic. In the Table C, we represent the number of clusters selected by the procedure on 100 resampling of the histogram.

Table A. Estimated parameters for the naive Gaussian mixture fit and the censored Gaussian mixture fits defined in Eq (B), for the datasets available in [2] and [3]. Note the consistency of the estimations, in particular in the partially and completely censored models.

Jones et al. [2]

Model	q_i	μ_1	σ_1	π_1	μ_2	σ_2	π_2	μ_3	σ_3	π_3
Naive		20.4	3.74	0.34	29.4	2.81	0.52	34.3	0.89	0.14
Partially	0.2	20.1	3.60	0.32	29.4	2.96	0.53	34.8	1.32	0.14
Completely		20.1	3.60	0.33	29.4	3.02	0.54	34.8	1.31	0.13

Cabrera et al. [3]

Model	q_i	μ_1	σ_1	π_1	μ_2	σ_2	π_2	μ_3	σ_3	π_3
Naive		19.8	2.05	0.20	25.6	2.99	0.39	34.3	2.36	0.40
Partially	0.4	20.2	2.19	0.26	26.0	2.58	0.43	34.5	2.66	0.41
Completely		20.6	3.45	0.31	26.3	2.11	0.24	34.4	2.98	0.43

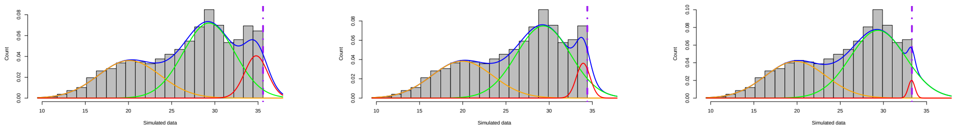

Fig D. Density of the fits of the censored model with three components (obtained when erasing data to the right of the threshold) with a threshold at 35.6 (left), 34.4 (middle) and 33.2 (right): the orange, green and red lines represent the density of each component and the blue line the density of the mixture. The histogram correspond to the one presented in [2].

Table B. Estimated parameters for the censored Gaussian mixture fit define in Eq (B) for different values of the threshold d_{cens}, applied to reconstructed data data with same distribution as in [2] erased above d_{cens}.

d_{cens}	μ_1	σ_1	π_1	μ_2	σ_2	π_2	μ_3	σ_3	π_3
35.6	20.13	3.60	0.33	29.41	3.02	0.54	34.81	1.31	0.13
34.4	20.13	3.61	0.35	29.35	2.99	0.57	34.21	1.03	0.08
33.2	19.97	3.56	0.03	29.40	3.14	0.59	33.21	1.16	0.48

Table C. Repartition of the number of clusters according to the considered symptomatic or asymptomatic dataset in [4].

	Clusters	
	2	3
Symptomatic	33%	67%
Asymptomatic	0%	100%

For the symptomatic population, the 3 cluster decomposition is selected twice more often than 2 cluster one. For the asymptomatic population, 3 clusters were selected for every resampling. In the main text, we consider a 3 cluster decomposition for both datasets.

In Fig E, we represents the estimations the mixture densities for each distribution.

For the censored mixture, we obtain the following estimations (see table D and Fig F).

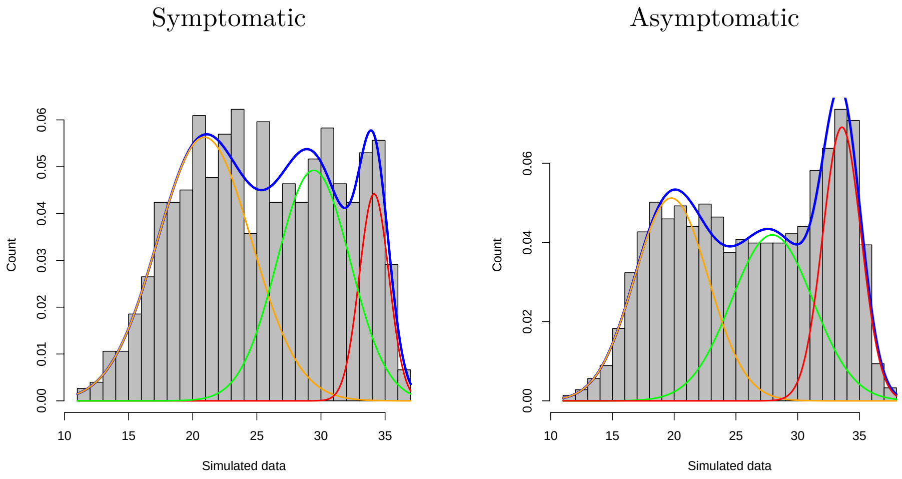

Fig E. Representation of the histogram for each distribution (symptomatic on left and asymptomatic on right) with the estimation of the mixture densities.

Table D. Estimated parameters for the censored Gaussian mixture fit define in Eq (B) for different values of the threshold d_{cens}, applied to reconstructed data data with same distribution as in Lennon at al. [4] erased above d_{cens}.

	d_{cens}	μ_1	σ_1	π_1	μ_2	σ_2	π_2	μ_3	σ_3	π_3
sympto	36	19.98	3.41	0.36	26.68	4.48	0.38	33.5	3.3	0.24
	35	20.41	3.47	0.46	29.59	4.15	0.45	92.01	10.09	0.07
	34	19.7	3.28	0.36	26.16	4.39	0.41	34.96	4.58	0.22
	33	19.03	3.08	0.32	24.9	3.71	0.45	31.53	2.66	0.22
asympto	37	19.24	2.81	0.31	27.41	4.23	0.43	33.56	1.59	0.24
	36	19.19	2.79	0.3	27.45	4.66	0.46	33.68	1.78	0.23
	35	18.47	2.49	0.23	24.3	3.77	0.38	34.83	3.74	0.37
	34	18.75	2.6	0.28	25.35	4.03	0.44	34.76	3.15	0.26

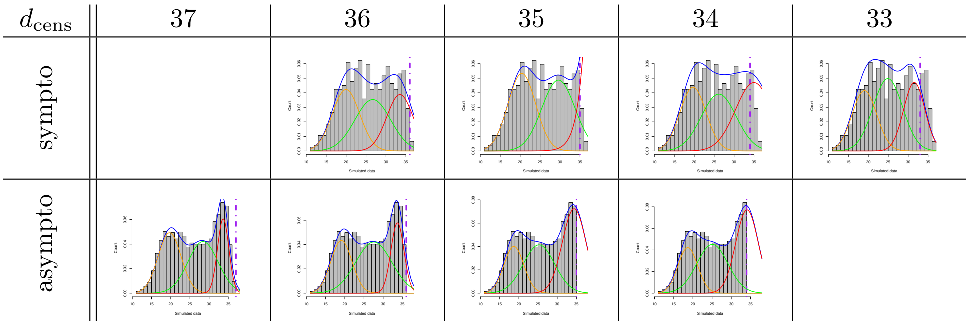

Fig F. Density of the fits of the censored model with three components (obtained when erasing data to the right of the threshold) with a threshold (columns) for the two datasets (rows): the orange, green and red lines represent the density of each component and the blue line the density of the mixture. The histogram correspond to the one presented in Lennon et al. [4].

For the censored mixture, we obtain the following estimations (see table E and Fig G).

Table E. Estimated parameters for the partially censored Gaussian mixture fit define in Eq (B) for different values of the threshold d_{cens}, applied to reconstructed data data with same distribution as in Lennon et al. [4] erased above d_{cens}.

	d_{cens}	q_i	μ_1	σ_1	π_1	μ_2	σ_2	π_2	μ_3	σ_3	π_3
symp	36	0.39	20.6	3.52	0.47	29.59	3.61	0.43	34.26	1.18	0.09
	35	0.76	21.28	3.8	0.55	30.26	2.82	0.34	34.54	0.96	0.09
asymp	37	0.51	19.55	2.93	0.35	27.8	3.75	0.38	33.56	1.6	0.25
	36	0.41	19.53	2.91	0.35	27.79	3.82	0.38	33.67	1.74	0.25
	35	0.82	19.64	2.96	0.36	28.01	3.62	0.37	33.67	1.6	0.25

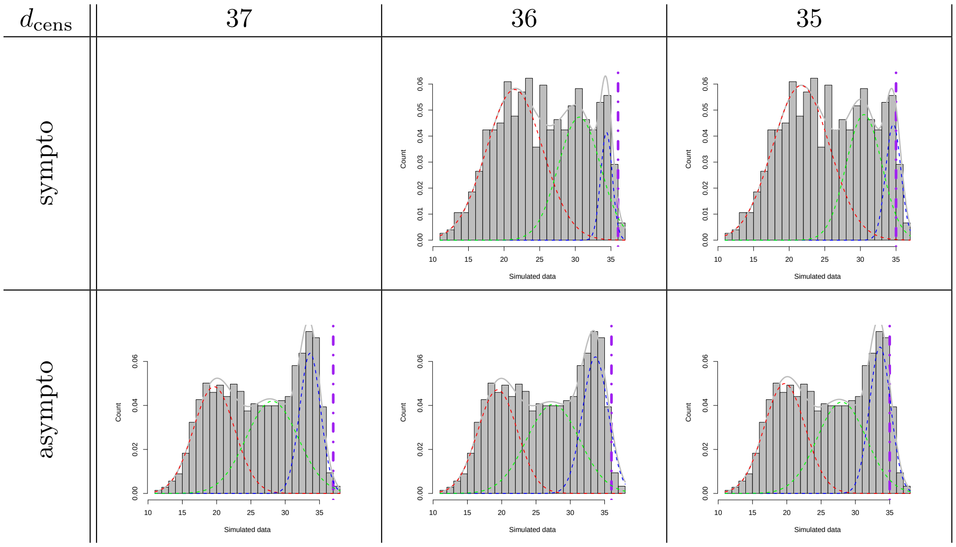

Fig G. Density of the fits of the partially censored model with three components (obtained when erasing data to the right of the threshold) with a threshold (columns) for the two datasets (rows): the orange, green and red lines represent the density of each component and the blue line the density of the mixture. The histogram correspond to the one presented in Lennon et al. [4]

I.4 Analysis of the ImpactSaliva dataset (Watkins et al. [5])

For the censure data, we refer to Table F and Fig H. For the partially censored, we refer to Table G and the Fig I.

Table F. Estimated parameters for the censored Gaussian mixture fit define in Eq (B) for different values of the threshold d_{cens}, applied to reconstructed data data with same distribution as in the ImpactSaliva (Watkins et al. [5]) dataset, erased above d_{cens}.

d_{cens}	μ_1	σ_1	π_1	μ_2	σ_2	π_2
44	30.8	6.82	0.86	40	0.47	0.13
43	31.19	7.1	0.87	40.02	0.45	0.12
42	30.79	6.83	0.86	40	0.47	0.13
41	31.19	7.09	0.86	40.05	0.52	0.13
40	31.67	7.37	0.96	39.49	0.02	0.03

d_{cens}				
37	36	35	34	33

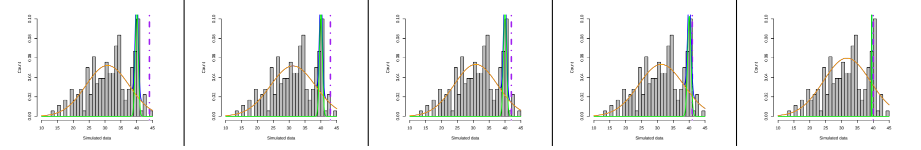

Fig H. Density of the fits of the censored model with two components (obtained when erasing data to the right of the threshold) with a threshold (columns) for the two datasets (rows): the orange and green lines represent the density of each component and the blue line the density of the mixture. The histogram correspond to the one presented in ImpactSaliva dataset [5].

Table G. Estimated parameters for the partially censored Gaussian mixture fit define in Eq (B) for different values of the threshold d_{cens}, applied to reconstructed data data with same distribution as in the ImpactSaliva dataset [5] erased above d_{cens}.

d_{cens}	q_i	μ_1	σ_1	π_1	μ_2	σ_2	π_2
44	0.95	30.74	6.75	0.86	40	0.47	0.13
43	0.95	31.13	7.04	0.87	40.01	0.46	0.12
42	0.76	30.46	6.5	0.86	39.99	0.48	0.13
41	0.08	30.69	6.68	0.86	40.05	0.6	0.13

d_{cens}			
37	36	35	34

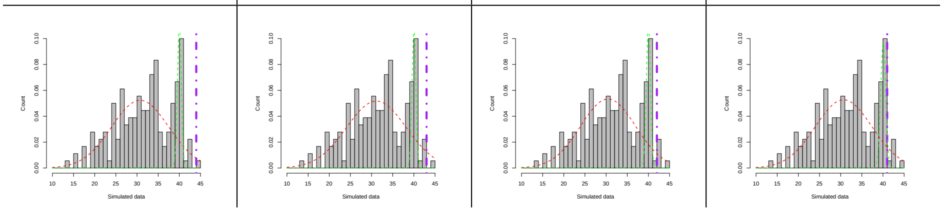

Fig I. Density of the fits of the partially censored model with two components (obtained when erasing data to the right of the threshold) with a threshold (columns) for the two datasets (rows): the orange and green lines represent the density of each component and the blue line the density of the mixture. The histogram corresponds to the ImpactSaliva dataset.

II Estimation of the false-negative risk in the presence of multiple positive individuals in the pool

We treat here the case of a pool of N samples that contains $k > 1$ positive individuals. We also consider the risk of defective sampling (e.g. that the swabs fails to collect viral load in an infected individual), which we denote ζ. The probability of having a negative pool result given that there is k positive samples within the pool reads, according to the model presented in Eq (4):

$$\mathbb{P}\left[-|k+\right] = \sum_{j=1}^{k} \binom{k}{j} \zeta^{k-j}(1-\zeta)^j \mathbb{P}\left[\log_2\left(\sum_{i=1,\dots,j} C_i/N\right) > d_{\text{cens}}\right]. \tag{D}$$

Under the two assumptions that:

1. the viral load distribution spans several order of magnitudes (e.g. log-normal distributed), so that, following Eq (7):

$$\mathbb{P}\left[\log_2\left(\sum_{i=1,\dots,j} C_i/N\right) > d_{\max}\right] = \mathbb{P}\left[\min_{i=1,\dots,j}(\log_2(C_i)) > d_{\max}^{(N)}\right], \tag{E}$$

   with $d_{\max}^{(N)} = d_{\text{cens}} - \log_2(N)$.

2. the viral loads between the k infected individuals are independent, in which case:

$$\mathbb{P}\left[\min_{i=1,\dots,j}(\log_2(C_i)) > d_{\max}^{(N)}\right] = \mathbb{P}\left[\log_2(C_1) > d_{\max}^{(N)})\right]^j, \tag{F}$$

we find that Eq (D) takes the simple expression:

$$\mathbb{P}\left[-|k+\right] = \left(\zeta + (1-\zeta)(1-\mathbb{P}\left[\log_2(C_1) < d_{\max}^{(N)})\right]\right)^k. \tag{G}$$

In Fig J, in the case of correlated samples, we find that the false negative risk in pooling is greatly reduced if there is more than one positive sample in the pool. The origin of such false-negative reduction is the large variability in viral load and the fact that the amplification technique is particularly sensitive to the highest viral load in the sample. Such false-negative reduction is robust to the presence of a finite risk of defective sampling $\zeta = 5\%$.

In addition, one may expect the number of positive k to be distributed according to a binomial distribution with a parameter p corresponding to the prevalence of the disease. Conditioned on the probability that there is at least one individual that is infected within the pool, the conditional probability that $k \geq 1$ is the number of infected individuals then reads

$$\mathbb{P}\left[k+|+\right] = \frac{1}{1-(1-p)^N}\binom{N}{k} p^k (1-p)^{N-k}, \tag{H}$$

which leads to the following expression for the averaged probability that the pool test turns negative although there is at least one positive individuals in the community

$$\begin{aligned}
&\mathbb{P}\left[-|+\right] \\
=&\frac{1}{1-(1-p)^N}\sum_{k=1}^{N}\binom{N}{k} p^k(1-p)^{N-k}\left(\zeta + (1-\zeta)(1-\mathbb{P}\left[\log_2(C_1) < d_{\max}^{(N)})\right]\right)^k, &\text{(I)}\\
=&\frac{1}{1-(1-p)^N}\left\{\left(p\left(\zeta + (1-\zeta)(1-\mathbb{P}\left[\log_2(C_1) < d_{\max}^{(N)})\right]\right) + 1 - p\right)^N - (1-p)^N\right\}. &\text{(J)}
\end{aligned}$$

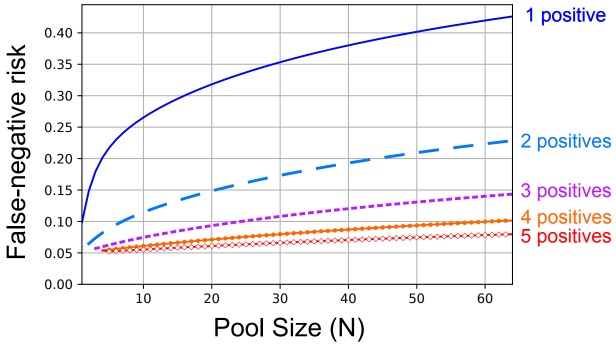

Fig J. Evaluation of the total risk of false negatives estimated according to Eq (D) as a function of the pool size N for several values of the number of positive samples in the pool $k = 1$ (blue solid line); $k = 2$ (cyan dashed line); $k = 3$ (magenta line); $k = 4$ (diamond orange line); $k = 5$ (circle red line). We consider a risk that the sample is defective $\zeta = 0.05$.

As shown in Fig K, the averaged false-negative probability risk is not necessarily a monotonous function.

A similar non-linear relation between the false-negative rate and the underlying population prevalence is also reported in [6].

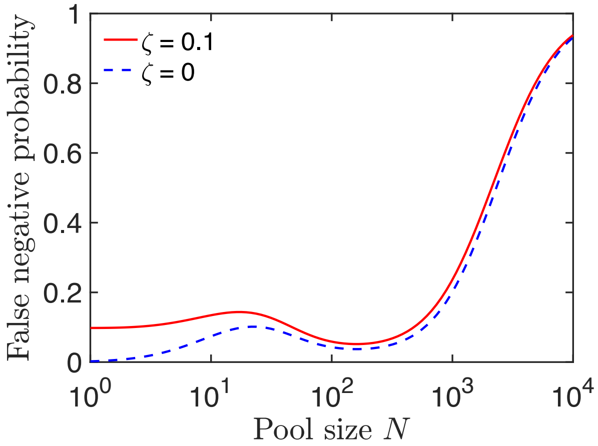

Fig K. Example of a counter-intuitive evolution in averaged community false negative risk of false negatives, as defined through Eq (J), as a function of the pool size N (solid red curve) with a defective sampling probability $\zeta = 0.10$. (dashed blue curve) without defective sampling., considering a single Gaussian distribution of viral loads with $\mu = 27$ and $\sigma = 2$.

III Measuring the prevalence with ideal tests

We present here some of results obtained from the computations made in Sec III, where we assumed perfect group testing and used it to measure prevalence in the population. Note that with a perfect test, the question of early detection of an outbreak in a community becomes much simpler : one just need to test everyone at regular time intervals with a single test.

III.1 Proof of the confidence intervals for the prevalence measurement

We recapitulate a derivation that closely follows the one of a seminal paper, [7]. We assume that we have n tests at our disposal. Given $N \in \mathbb{N}$, we sample nN individuals at random in the general population, and organize n pools of N individuals. Each of these pools is then tested using the perfect tests. For all $i \leq n$, we write $X_i^{(N)} = 1$ if the ith test is positive (i.e. if and only if at least one of the N individuals in the ith pool is infected), and $X_i^{(N)} = 0$ otherwise. We denote by p the (unknown) proportion of infected individuals in the population, then $(X_i^{(N)}, i \leq n)$ forms an independent and identically distributed (i.i.d.) sequence of Bernoulli random variables with parameter $1-(1-p)^N$.

Lemma III.1. *Writing $\overline{X}_n^{(N)} = \frac{1}{n}\sum_{j=1}^N X_j^{(N)}$, the quantity $1-(1-\overline{X}_n^{(N)})^{1/N}$ is a strongly consistent and asymptotically normal estimator of p. A confidence interval of asymptotic level $1-\alpha$ is*

$$\mathrm{CI}_{1-\alpha}(p) = \left[1-(1-\overline{X}_n^{(N)})^{1/N} \pm \frac{q_\alpha(1-\overline{X}_n^{(N)})^{1/N-1}\sqrt{\overline{X}_n^{(N)}(1-\overline{X}_n^{(N)})}}{\sqrt{n}N}\right], \tag{K}$$

where q_α is the quantile of order $1-\alpha/2$ of the standard Gaussian random variable.

Proof. Note that $(X_j^{(N)}, j \leq n)$ is a standard Bernoulli model, hence $\overline{X}_n^{(N)}$ is a consistent and asymptotically normal estimator of $f(p) = 1-(1-p)^N$. Hence, using that f^{-1} is $\mathcal{C}^1$ and Slutsky's lemma, we deduce all the above properties of the estimator $f^{-1}(\overline{X}_n^{(N)})$ of p. □

Remark III.2. As $\lim_{n\to\infty} 1-(1-\overline{X}_n^{(N)})^{1/N} = p$ almost surely, for any $N \in \mathbb{N}$ the width of the confidence interval defined in Lemma III.1 satisfies

$$\frac{2q_\alpha(1-\overline{X}_n^{(N)})^{1/N-1}\sqrt{\overline{X}_n^{(N)}(1-\overline{X}_n^{(N)})}}{\sqrt{n}N} \underset{n\to\infty}{\sim} \frac{2q_\alpha}{\sqrt{n}}\frac{(1-p)}{N}\sqrt{\frac{1-(1-p)^N}{(1-p)^N}} \quad \text{a.s.} \tag{L}$$

III.2 Proof of the optimal size for the prevalence measurement

The width of the confidence interval defined in Eq (K) behaves asymptotically, by law of large numbers, as $2(1-p)q_\alpha f(p,N)/\sqrt{n}$ where

$$f(p,n,N) := \frac{1}{N}\cdot\sqrt{\frac{1-(1-p)^N}{(1-p)^N}}.$$

An optimal choice for the value of N given p can thus be chosen as the value of N minimizing $f(p,\cdot)$. Indeed, this choice minimizes the width of the confidence interval for

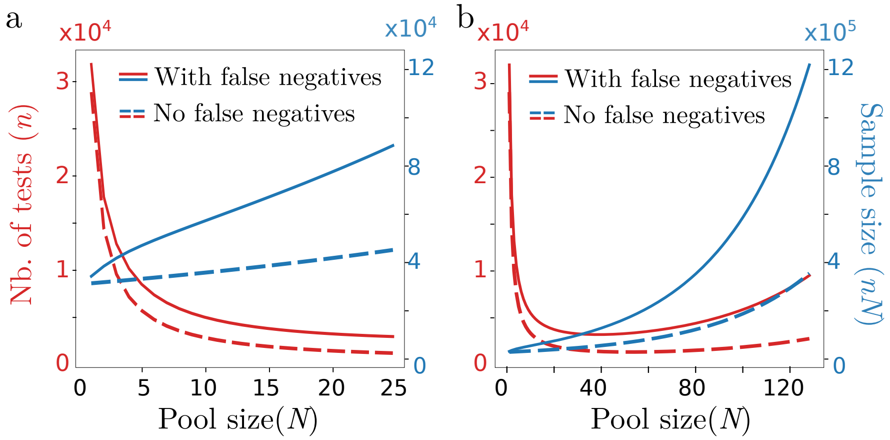

Fig L. (A,B) Total number of tests (red) and total number of sampled individuals (blue) in order to estimate a prevalence of $p = 3\%$ with a $\pm 0.2\%$ precision with 95% confidence interval as a function of the pool size N for the perfect case with no false negative (dashed lines) versus the case with false negatives (solid lines) estimated according to the Jones et al. dataset [2]. In (A) N ranges from 0 to 25; in (B) N ranges from 0 to 128; as visible in (B), the valley around the optimal pool size $N_{\text{opt}}^{(\text{perf})} \approx 50$ is large: near optimal savings in tests are achieved even for moderately large pool sizes that require smaller number of individuals to sample.

the measured prevalence. Plots of $N \mapsto f(p, N)$ are provided for several values of p in Figure M.

We observe that the quantity N^{opt} will approach the quantity x^* which minimizes $x \mapsto \log f(p, x)$. Observe that x^* then satisfies

$$0 = -\frac{1}{x^*} + \frac{1}{2}\frac{-\log(1-p)}{1 - e^{x^* \log(1-p)}} \iff x^*(-\log(1-p)) = 2(1 - e^{x^* \log(1-p)})$$
$$\iff (x^*(-\log(1-p)) - 2)\, e^{x^*(-\log(1-p))} = -2$$

Therefore, the minimum of $x \mapsto f(p, x)$ is attained at point

$$x^* = \frac{2 + W(-2e^{-2})}{-\log(1-p)},$$

with W the Lambert W function (the inverse function of $x \mapsto xe^x$).

Observe that different optimization could be considered, for example choosing values of n and N that minimize the width of the interval of confidence on the measure of the prevalence for a given cost C, measured as $aN + nN$, with a representing the cost of a test, the cost of sampling an individual being normalized at 1. In this situation, the optimization problem becomes very similar, using that $n = C/N - a$. In this situation, the width of the asymptotic confidence interval decays as $2(1-p)q_\alpha g(p, C, N)$ with

$$g(p, C, N) = \frac{1}{\sqrt{CN - aN^2}} \sqrt{\frac{1 - (1-p)^N}{(1-p)^N}}.$$

The optimal value of N in this situation interpolates between $N = 1$ when $a \to 0$ and $N = x^*$ when $a \to \infty$.

III.3 Number of tests and sample size as function of the population prevalence

We trace here, for various values of the prevalence, the number of tests and total number of samples needed to archive a given precision for the confidence interval. We observe that over a large range of prevalences, the number of tests needed to reach a given precision on the measure of the prevalence remains small for a large range of pool sizes. On the other hand, the total number of individuals to sample grows quadratically, and the test sensibility decreases with the size of the pools. Hence, it might be interesting to consider a suboptimal choices $N < N_{\text{opt}}$ for the pool sizes when measuring the prevalence.

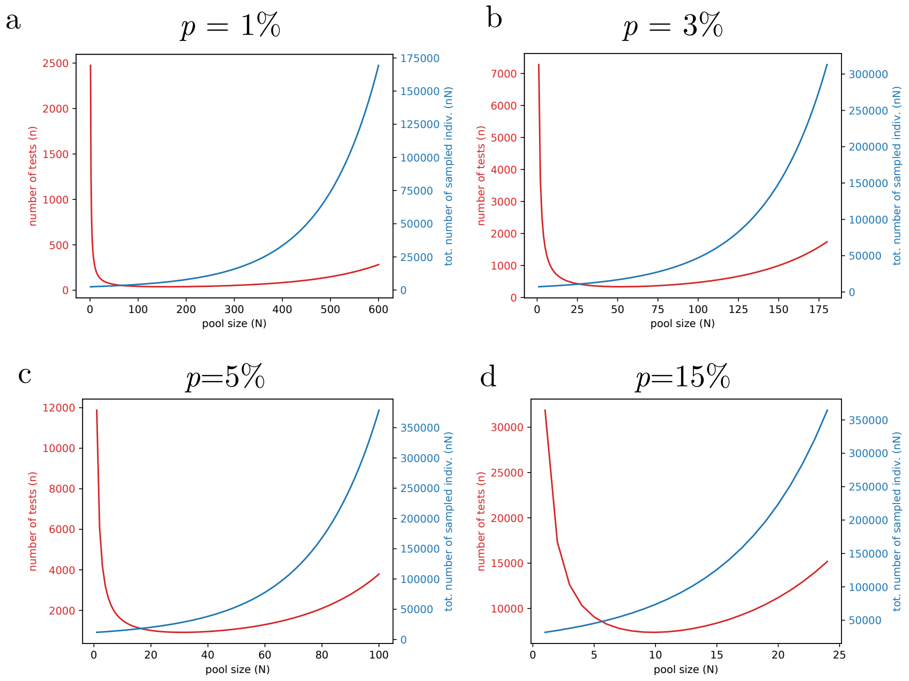

Fig M. Total number of tests and sampled individuals so that the width of the 95% confidence interval is smaller than 0.4% as a function of the pool size N chosen for a perfect test, for a prevalence p equal to $p = 1\%$ (A), $p = 3\%$ (B), $p = 5\%$ (C), $p = 15\%$ (D).

III.4 Bayesian inference

We are now interested in a Bayesian approach to the measure of prevalence. We started with an initial prior distribution with density $f_0(p) = 6p(1-p)\mathbf{1}_{\{0\le p\le 1\}}$ for the prevalence, and for each new test j we do the following:

1. take the the mean value $\overline{p}_{j-1} = \int_0^1 p f_{j-1}(p)\mathrm{d}p$ of the prior;
2. choose the size N_j of the pool of the jth test computed as (cf. Eq (18)):

$$N_j = \left\lfloor -\frac{c_\star}{\log(1-\overline{p}_{j-1})} \right\rfloor; \tag{M}$$

3. choose N_j individuals at random and test them in a group:
   - if the test is positive, then $f_j(p) = C_j^+(1-(1-p)^{N_j})f_{j-1}(p)$;

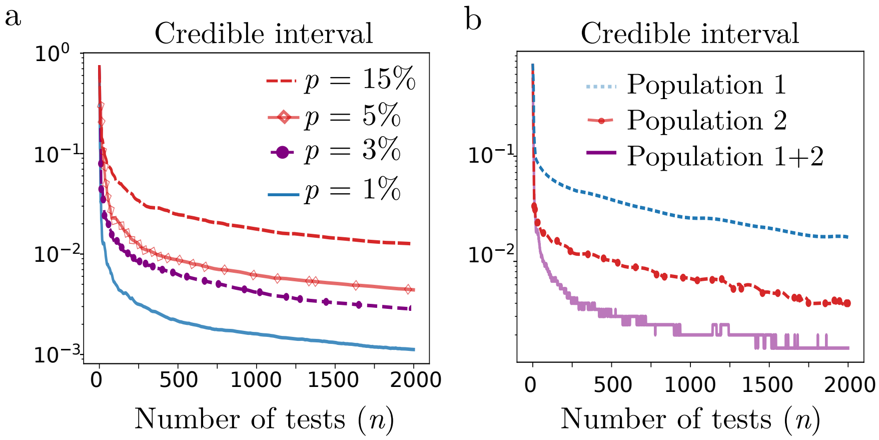

Fig N. (A) Width of the 95% credible interval on the prevalance p with adaptative Bayesian sampling as a function of the number of tests n for a set of values in the prevalence ranging from $p = 15\%$ (top, magenta dashed line) to $p = 1\%$ (bottom, blue solid line). (B) Width of the credible intervals in a two-category mixed population for the prevalence either: in the general population (magenta solid line); for the less exposed population 1 with a prevalence of 0.5%, representing 80% of the general population (blue dashed line); for the more at-risk population 2 with a prevalence of 5% representing 20% of the general population (red dotted line with circles).

- if the test is negative, then $f_j(p) = C_j^-(1-p)^{N_j} f_{j-1}(p)$;

with $C_j^{\pm}$ normalizing constants, chosen such that $\int_0^1 f_j(p)\mathrm{d}p = 1$.

We trace in Fig N the result in blue of this experiment, the 95% credible interval being $[a_j, b_j]$, with a_j being the 2.5%th quantile of f_j and b_j its 97.5% quantile.

Simultaneously to this statistical experiment, one can follow the prevalence in sub-populations of interest. For example, if we assume the population consists of two sub-populations 1 and 2 with different prevalences p_1 and p_2. Starting with a prior distribution $f_j(p_1, p_2)\mathrm{d}p_1\mathrm{d}p_2$ for these prevalences, if a group consisting of a individuals of the first sub-population and b individuals of the second population is sampled positive, then Bayes rules gives $C_{j+1}^+(1-(1-p_1)^a(1-p_2)^b)$ for the updated law of (p_1, p_2). A similar update is made if the test is negative. As a result, we get estimates for the prevalence in each sub-population at the same time as we are measuring the prevalence in the overall population.

We test the above statistical experiment on a population which is composed of two sub-populations, one large subpopulation of sparsely exposed individuals (prevalence 0.5%, representing 4/5th of the whole population), and a smaller subpopulation of very exposed individuals (prevalence 5%). At each step, we choose the size of the pool according to the available estimate for the prevalence in the complete population. The composition of the pool in terms of individuals of each sub-population is chosen at random (at the jth step, there are $\mathrm{Ber}(N_j, 0.8)$ individuals of the first sub-population). We also update our estimation of the prevalences (p_1, p_2) in each of the two sub-populations.

The results are traced in Fig N in orange and green curves. One can see that the width of the credibility intervals of the sub-populations decay much slower than for the whole population. The reason is that the size of the groups are optimized to measure as precisely as possible the mean value p.

However, observe that even with a naive group construction (without segregating individuals according to their sub-population), one can extract information on the

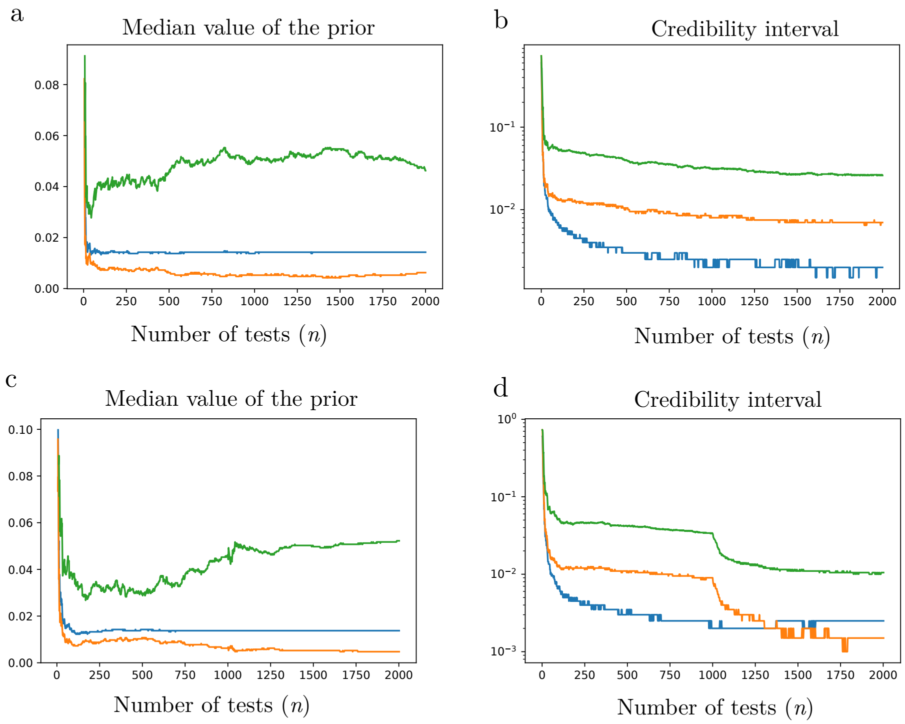

Fig O. (A-B) Bayesian estimation of the parameters of a mixed population, consisting of 80% individuals of type 1 with a prevalence of 0.5% and 20% individuals of type 2 with a prevalence of 5%. Pooled samples are constituted by sampling randomly individuals from the two sub-populations, with a size optimized for the speed of convergence of the overall prevalence of 1.4%. (A-C) Median value of the priors, overall population in blue, first resp. second population in orange resp. green. (B-D) Width of the 95% credible intervals. In (C,D), the first 1000 tests are made on groups whose size is optimized to estimate the prevalence of the overall population, the next 1000 tests are divided into two groups that are used on homogeneous sets of the sub-populations, in groups optimized to estimate the prevalences within these sub-populations. This has the effect of drastically improving the speed of convergence of the estimator of the prevalence in the subpopulations.

prevalence of the sub-populations of interest. Therefore, a design for the measure of the prevalence in a stratified population could be the following: in a first time, pool testing is implemented on randomly constructed group of individuals from the general population. Data is then analysed to detect sub-populations with different prevalences (e.g. according to geography, age, occupation, ...). In a second time, once sub-populations of interest are identified, pool testing is applied to each of the sub-populations independently. We implemented this method if Fig O, with the same number of tests a much more detailed estimate of the prevalence is obtained.

IV Optimization of the frequency of test

We study here the impact of the regularity of tests on the rate of detection of a infection occurring in a closed community of A individuals. We assume a fixed budget of tests per unit of time, which are made on pools of fixed size N of individuals chosen at random in the community. We compare different strategies for the detection of outbreaks in the community depending on the frequency of the tests. At one extreme, a single test is made on one pool of N individuals every T units of time, at the other extreme, every individual in the community is tested every TA/N unit of time. These two strategies both use on average one test every T units of time, the first one emphasizing the regularity of testing, while the second one exhaustively tests every member of the community.

More generally, for all $1 \leq f \leq A/N$, we can consider the detection strategy with period f in which every fT units of time, a number fN of individuals in the community get tested in pooled samples. The aim of this strategy is to detect as soon as possible the infection of the community in order to deploy additional aseptic measures and prepare for a potential influx of hospitalized patients from this community. Perhaps unsurprisingly, we show that the smaller the frequency is, the lower the number of infected individuals is at the first time of detection of the outbreak. However, note that in many closed communities (e.g. professional athletes in Germany football league, US baseball league, etc.) the opposite strategy is put in practice with testing of the whole team at regular intervals rather than randomly selected members every day.

We model the initial outbreak in a community as a Crump-Mode-Jagers (CMJ) process [8]. Individuals are infected from the outside at a Poisson rate of small parameter η. Every infected individual then goes through several stages of the disease. After an incubation period t_O, the individual starts excreting the virus up to the time t_f. During that phase, the individual will infect members of its community at a Poisson rate of parameter λ. The probability that an individual eventually becomes symptomatic is denoted r; in this case, at a random time denoted S between t_O and t_f, the individual will start showing symptomatic.

For our purpose, the detection of the outbreak corresponds to the first time at which either:

- an individual becomes symptomatic in the population,
- or a pooled test turns positive.

We place ourselves in a stationary regime, with a screening strategy of period f. In this situation, as exterior infections happen according to a Poisson process, the first exterior infection will occur at a time chosen uniformly at random between two screening times. In other words, the first screening which might detect the outbreak will happen UfT units of time aft after the exterior infection. Comparing with the screening strategy with period 1, we see that the latter strategy will on average use $fT/2$ tests on the population between the first screening time of the strategy f. Additionally, the longer the time between the infection and the test, the higher the probability that a first symptomatic individual will appear, making useless the screening.

To quantify the above heuristic, we considered a simpler model in which $t_O = 0$ (the time of incubation is neglected), $t_f = \infty$ (the time of recovery is neglected) and the time of apparition of symptom S is chosen as an exponential random variable with parameter ρ. In this situation, we obtain analytic values for the number of infected individuals at the first detection time.

In this simplified model, the number of infected individuals t units of time after the first infection, denoted by $N(t)$ follows a standard Yule process [9], therefore $N(t)$ is distributed as a geometric random variable with parameter $1 - e^{-\lambda t}$. Moreover, given $N(t)$, a new infection will occur at rate $\lambda N(t)$ while an individual will become

symptomatic at rate $\kappa r N(t)$, using that a fraction r of the population is symptomatic. In the absence of screening, the apparition time of first symptom can be expressed as

$$T_s = \sum_{j=1}^{G} e_j, \tag{N}$$

where G is the number of infections up to the first symptomatic one and $e_j$is time interval between the $j-1$th and jth infection events; we consider G to be geometrically distributed ($\mathbb{P}(G=k) = p^{k-1}(1-p)$) with parameter $p = (r\kappa)/(\lambda + r\kappa)$ and that the e_j are independent exponential random variables with parameter $j(\lambda + r\kappa)$ (since each newly infected individual contribute to the intra-community attack rate by a multiplicative factor $\lambda + r\kappa$).

Averaging Eq (N), we find that the average apparition time of first symptom $\langle T_s \rangle$ reads

$$\langle T_s \rangle = \frac{-\log(1 - \frac{\lambda}{\lambda + r\kappa})}{(\lambda + r\kappa)}. \tag{O}$$

Next, we observe that at the first screening time, there is a number $N(fTU)$ of infected individuals with U an independent uniform random variable. Based on Eq (14), we find that, as long as $e^{\lambda f T} \ll A$, the first screening test will detect the outbreak with a probability approximately equal to

$$\mathbb{P}\,[+] = \langle \Phi_0(d_{\text{cens}}^{(N)}) \left(1 - (1 - N(fTU)/A)^{fN}\right) \rangle, \tag{P}$$

$$\approx \Phi_0(d_{\text{cens}}^{(N)}) fN \langle N(fTU) \rangle / A, \tag{Q}$$

$$\approx \frac{\Phi_0(d_{\text{cens}}^{(N)}) N}{AT} (e^{\lambda f T} - 1). \tag{R}$$

where $1 - \Phi_0(d_{\text{cens}}^{(N)})$ is the group test false-negative rate. From Eq (R), the screening detection probability quantity is an increasing of the sampling period period T and infection rate f; the corresponding detection time will then exceeding the onset of symptom time $\langle T_s \rangle$ for large infection rates f or sampling periods T.

Factoring in the fact that the number of infected individuals grows exponentially fast, and that more frequent screening implies several chances of detecting the infection before first symptoms show up, these computations show that frequent testing is key to a successful screening strategy, much more than exhaustive testing of the community.

As a validation of the previous computations, we estimate by Monte-Carlo method the average number of infected individuals $\langle N(T_d) \rangle$, we find that a screening strategy consisting in sampling a random subgroup of the community as frequently as possible is more efficient than the one consisting in testing larger portion of the community at less frequent time intervals. In Fig P, we compare different screening scenarios for a large community composed of $A = 1000$ individuals. We vary the value of the screening time interval τ while keeping fixed (1) the average number of tests per unit of time and (2) the size of the pools on which each test is used. Our simulation range from checking N individuals every day (with one test) to checking $12 \times N$ individuals in 12 pools every 12 days.

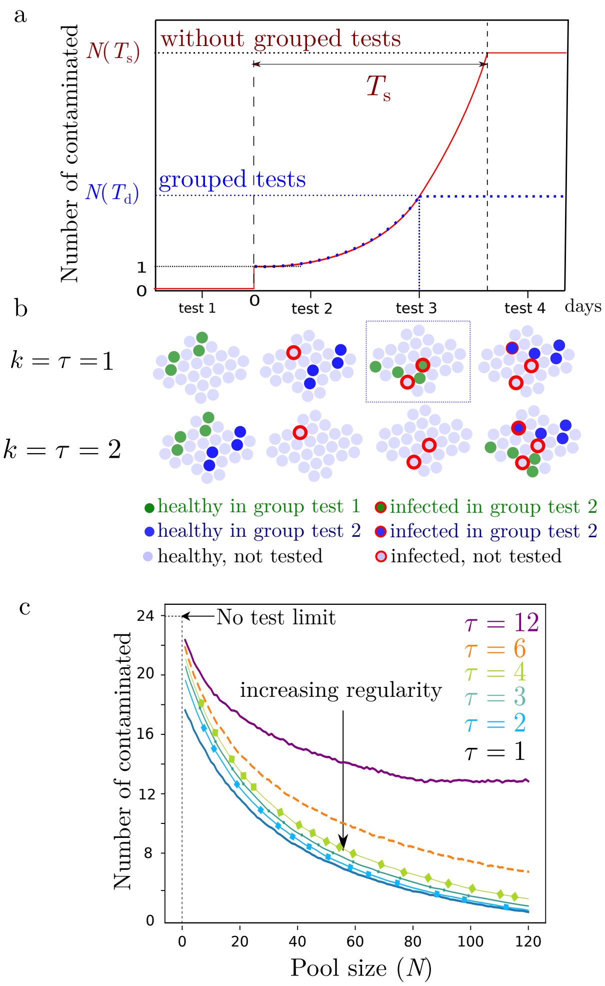

Fig P. (A) Sketch of the time evolution of the number of infected individuals in a community. The patient 0 is infected from the outside of the community 0.8 units of time after a test date. In the absence of screening tests, the infection is detected at the time $T = T_s$ (after appearance of the first symptoms); with grouped tests, an infected individual is detected at a time $T = T_d$. (B) Sketch of two group testing strategies, here with pools of size $N = 4$, one with a single ($k = 1$) grouped tests every day ($\tau = 1$); the other with $k = 2$ grouped tests every second day ($\tau = 2$); the second strategy (least frequent testing) fails to detect the outbreak early and results in more infections. (C) Number of infected individuals at the detection of the outbreak as a function of the pool size, using $k = \tau$ tests performed at τ-day intervals, with $\tau = 12$ (solid purple line), $\tau = 6$ (dashed orange line), $\tau = 4$ (dark green solid line with square), $\tau = 3$ (light green solid line with circles), $\tau = 2$ (cyan line with squares) and $\tau = 1$ (solid blue line). Here we consider a large community composed of $A = 1000$ individuals. The patient 0 has a viral load concentration distributed according to a log-normal distribution with mean $\mu_0 = 30$ and standard deviation $\sigma_0 = 2\log_2(2)$; all others parameters can be found in Table P.

Table H. Table with standard parameter values considered in Sec IV of S1 Text

Symbol	Meaning	Value
t_O	Incubation time (as defined in Eq (9))	0
t_f	End of symptom time (as defined in Eq (9))	∞
S	Random time of onset of symptoms (mean κ)	
κ	Mean onset of symptoms time	5 days
η	External attack rate on the community	$\eta \ll \lambda$
λ	Intra-community infection rate	$0.5\,\text{days}^{-1}$
r	Probability that an individual remains asymptomatic	40 %
τ	Time interval between grouped tests	$1 - 12$ days
A	Total number in the community	1000
N	Pool size	1–128

References

1. Nielsen OE. Information and exponential families : in statistical theory. Chichester U.K. New York: John Wiley & Sons; 2014.

2. Jones TC, Mühlemann B, Talitha V, Marta Z, Hofmann J, Stein A, et al. An analysis of SARS-CoV-2 viral load by patient age. Preprint Charité Hospital. 2020;.

3. Cabrera JJ, Rey S, Perez S, Martinez-Lamas L, Cores-Calvo O, Torres J, et al. Pooling for SARS-CoV-2 control in care institutions. medRxiv. 2020;doi:10.1101/2020.05.30.20108597.

4. Lennon NJ, Bhattacharyya RP, Mina MJ, Rehm HL, Hung DT, Smole S, et al. Comparison of viral levels in individuals with or without symptoms at time of COVID-19 testing among 32,480 residents and staff of nursing homes and assisted living facilities in Massachusetts. medRxiv. 2020; p. 2020.07.20.20157792.

5. Watkins AE, Fenichel EP, Weinberger DM, Vogels CBF, Brackney DE, Casanovas-Massana A, et al. Pooling saliva to increase SARS-CoV-2 testing capacity. medRxiv. 2020;doi:10.1101/2020.09.02.20183830.

6. Verwilt J, Mestdagh P, Vandesompele J. Evaluation of efficiency and sensitivity of 1D and 2D sample 1 pooling strategies for diagnostic screening purposes 2 3. medRxiv. 2020; p. 2020.07.17.20152702.

7. Thompson KH. Estimation of the Proportion of Vectors in a Natural Population of Insects. Biometrics. 1962;18(4):568. doi:10.2307/2527902.

8. Schertzer E, Simatos F. Height and contour processes of Crump-Mode-Jagers forests (I): general distribution and scaling limits in the case of short edges. Electron J Probab. 2018;23:43 pp. doi:10.1214/18-EJP151.

9. Meleard S. Modèles aléatoires en Ecologie et Evolution. CMAP; 2016. Available from: `http://www.cmap.polytechnique.fr/IMG/pdf/LIVRE07102013.pdf`.
