# Supplementary material for: Group testing as a strategy for COVID-19 epidemiological monitoring and community surveillance
Source: PLoS Comput Biol. 2021 Mar 4;17(3):e1008726. doi: 10.1371/journal.pcbi.1008726 (PMC7932094; doi:10.1371/journal.pcbi.1008726)
Supplement: Attachment — Submitted filename: ReviewersLetterPlosComp_11122020.pdf [file pcbi.1008726.s004.pdf]

Reviewer #1: Brault and colleagues present a computational framework for pooling samples prior to Sars-Cov-2 testing as a tool to estimate the prevalence of infection within a population. Pooling samples has often been used in the past to detect low-prevalence targets in an efficient manner by conserving tests, and therefore there has been great interest in adapting this to Covid19 testing. The unique insight here is that, in contrast to the usual approach, the authors do not examine pooled testing as a prelude to the testing of individual samples (i.e. for efficient eventual identification of infected individuals) but rather as an end in itself, in order to efficiently provide population surveillance. To do so, they develop a model of test-positivity grounded in apparently reasonable assumptions about test performance and viral load, estimate a simulated distribution of viral loads using a partially-published dataset, and use this to describe how their computational approaches can be adapted as a tool to monitor the appearance and tracking of prevalence within a defined population.

The major weakness of the paper, as the authors implicitly acknowledge, is the use of a simulated dataset of viral densities represented in Figure 3; this was estimated based upon inspection of a pre-print article reporting many thousands of results from Germany, but the exact data were not available for this report.

We can address some of the Reviewer 1 concerns in our new version of the manuscript.

First, we now challenge our prediction for the false-negative on the ImpactSaliva dataset from the published study [Watkins et al.], whereby the exact Cts values are provided. In that case, no data extraction nor simulation are needed (new Figure 4 page 14).

Second, we analyzed another histogram from [Lennon et al.] ; raw Cts are not provided and data extraction is performed. This data is the largest to date (to the best of our knowledge) to present separated Ct histograms between symptomatic or asymptomatic status of the individual at the time of testing.

As with previous datasets, we estimate the full robustness of our data estimation based on stochastic simulation regarding the distribution of Cts within each histogram bar. We obtained which lead us to consistent results with our previous analysis

We still discuss our results of the viral load distribution presented in [Jones et al.] and [Cabrera et al.] in the main and the Supplementary information, respectively. These datasets were the only one available at the time of submission.

Should the Reviewer think that we ought to include raw Cts from a large clinical dataset - I would like to mention that I have had one positive contact with the team behind the Jaafar et al. *Clinical Infectious Diseases* publication (which we did not include in the analysis). It might be possible for us to gain access to some raw Ct dataset within the next month(s).

We also mention that Jaafar et al. provides the exact number of individuals per Ct interval is provided; no plot data extraction would be involved. We could additionally include this dataset in our analysis.

Medical regulation appears to restrict disclosure of raw Ct values, hence, this would require extensive administrative work. In addition, we prefer to maintain the number of authors to the 3 of us - if possible.

Furthermore that source paper provides little information about how viral positivity and density are correlated with important clinical and demographic variables, such as age, symptomaticity, etc. Furthermore, what is the authors' hypothesis as to why these values are distributed as they are?

In the new version of the manuscript, we introduce a discussion regarding the interpretation of the observed multi-Gaussian viral distribution in terms of the epidemic stage - with a new paragraph: Sec. I.2.4. Interpretation of the Gaussian mixture model, page 9.

We implemented our Ct distribution analysis for 2 the populations of symptomatic and asymptomatic individuals from the preprint Lennon et al.

We think our interpretation shares some similarity with two recent preprints (which we cite):

(1) Ref. Hay et al. 10.1101/2020.10.08.20204222, which provides an evolution of the viral load within a (relatively large) hospital (Brigham Women's Hospital in Boston) across the epidemic first wave. The population-level viral load was weak during summer (low viral circulation periods).

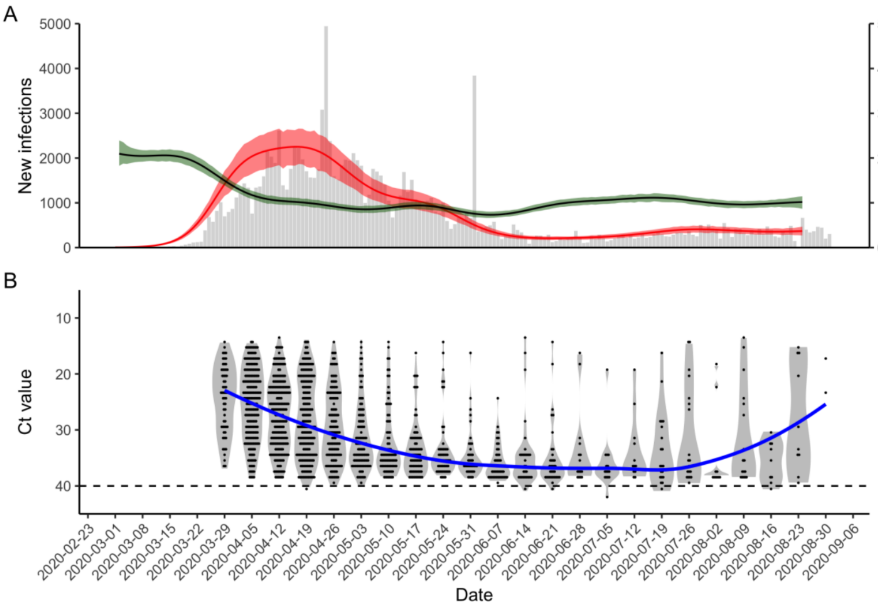

Figure 1 - (For convenience, we copy here the graph from Hay et al.) Correlation between the estimated number of daily new infections (red), effective reproductive number Rt (green) and measured viral load distribution in samples.

(2) Ref. Kiessler at al., which presents experimental data regarding the viral load evolution with time of symptomatic and asymptomatic individuals.

As this distribution evolves depending on the epidemic stage, our analysis has some important consequences for the interpretation of the rate of false negatives of asymptomatic individuals; the new dataset Lennon el al. is instrumental to achieve such interpretation.

Should we succeed in gaining access to a large-scale raw Ct values dataset (i.e. even larger than the 130 individuals ImpactSaliva dataset), we would be able to perform a more refined analysis of the Ct data with the epidemic stage; however, we think such quantitative epidemiological analysis goes beyond the methodology & mathematical scope of the current manuscript.

We are confident that we can already provide a satisfactory interpretation of the shape of the observed Ct distribution based on simple assumptions and publicly available histograms on the number of new cases. We think the added interpretation considerably strengthened our manuscript and we thank the reviewer for mentioning this point.

This has important implications for the applicability of their suggested testing protocols later in the report.

Following the reviewer comment, we now discuss the role of the viral load evolution and the interpretation of the censored Gaussian analysis in the epidemic outbreak surveillance section (previously Sec. IV, now Sec. II - page 15) within the new version of the manuscript.

Depending on the availability of tests, we propose two specific scenarios for the surveillance protocol :

1) if infection spreading is critically risky (e.g. among staff of a nursing home, let's say), we may opt for a Ct distribution that models the one of presymptomatic patient 0; based on our new model for the time evolution of the viral load, we indeed expect such distribution to differ from the one of the general population. Focusing on the case of early pre-symptomatic individuals also provides a lower bound on the efficiency of pool testing.

2) if testing is less accessible, only a fraction of the community is to be tested each week. It appears reasonable to consider that the viral load among a silent nascent cluster (which, potentially, could be one or two weeks old) could be deduced from our censored Gaussian analysis for asymptomatic individuals.

It would be helpful in the Introduction for the Authors to more clearly differentiate their goals from those of others' in the increasingly crowded covid-sample-pooling space. Namely that mentioned above, which is that the goal here is not to provide a tool to optimize pooled "cascade" testing and efficiently find the few infected individuals...

We thank the reviewer for this suggestion -- we changed our Introduction for the Authors accordingly, but also a large portion of our Introduction section.

As recognized by the reviewer, our paper is indeed not addressing the question of individual diagnosis (which has been the objective of numerous papers) but really the one of screening and surveillance --- and we now state this explicitly in the introduction.

… but rather for routine population surveillance , presumably to provide a metric by which to deploy population-level controls (i.e. distancing, etc).

We agree with Reviewer 1's comment regarding the interest of our work in defining a metric.

Institutions may consider using the prevalence as a scale in a predefined graded response scheme - e.g. in a college/university campus context, a rising prevalence could serve as a metric to decide whether collective sports events may or may not take place, or, at higher levels, whether to switch to remote teaching.

One of the 3 main objectives of our work is to derive confidence intervals on the point prevalence based on pool positivity results. In the context of a community surveillance, our framework can help to identify early signs of a spreading event with the community from a stochastic, background fluctuation in the number of cases (e.g. due to a constant external attack rate on the community). Mid-October, Cornell University pool surveillance program detected an increase in the point prevalence. Such increase appeared to be significantly higher than the average variability in the measured prevalence within the previous weeks; such increase was allegedly related to an unmasked student gathering.

We have added within the introduction, page 2, some elements of contexts inspired by the implementation of pool testing strategies in university campuses across the United States and in the UK (Nottingham & Cambridge Universities).

Describing a roadmap for how these data might be ultimately actionable would sharpen this focus and clarify the distinct aims of this effort.

Following the referee suggestion, we have added a road map/plan of action paragraph that is directly inspired by our proposal for surveillance of a campus in France. This roadmap is based on realistic technical and financial constraints. The added paragraph is to be found Sec. II.2. Risk mitigation from a single pre-symptomatic individual, page 15, within the main text.

Very minor commments: "Contaminated" is an odd choice and I suggest infected in its place. Also viral "charge" is non-standard to me, and "load" would be a more common usage.

Such misuse is explained by the fact that we were “contaminated” by the French terminology (*contaminé* stands for *infected* and *charge virale* for *viral load*)

We have replaced all occurrences of *contaminated* by infected; same for *charge* by load.

We thank the reviewer for her/his helpful comments.

Reviewer #2: The authors present a modeling study of pooled testing for SARS-CoV-2. The article is not framed well in infectious disease epidemiology and I recommend the authors work with an infectious disease epidemiologist before resubmitting their paper for review.

We followed the reviewer 2 suggestion and had a critical proofreading from an infectious disease epidemiologist. In addition, we sought for additional proofreading from the French MODCOV modelling initiative; we consulted the:

- epidemiologist Pr. Catherine Hill
- molecular biologist and CNRS Director Françoise Praz,

We thank the reviewer for his suggestions of reframing our manuscript so as to convey a more relevant message.

Many of the ideas that the authors present have been discussed at great length in the field of infectious disease epidemiology, particularly around infectious disease surveillance.

We understand that the former framing of the introduction may have appeared odd and unadapted.

Following the reviewer suggestion, we have significantly rephrased our introduction. We insist on the interest of pooling to screen large populations and to break chains of transmissions.

4. There is great value in estimating the false negativity rate among pooled testing. I would have liked to see that particular problem highlighted and improved, with modeling taking into account an estimate of actual prevalence at the time of testing.

We shift the discussion of point 4 to the beginning of this response.

We thank the reviewer for recognizing the value of our work on the prediction of the false-negative rates:

1) We added a new Figure 4 (page 14) for the estimated false negative rate based on the individual Ct values; our analysis correctly fit to the clinical estimation of the false-negative rates for 3 pool sizes (N = 5, 10, 20) [Watkins et al.].

2) We also put forward our theoretical analysis of the “hitchhiking” effect, which had been introduced at the end of the Supplementary Section. The term “hitchhiking” effect is coined in Barak et al, where correlations in the infectious status in pools (usually composed of individuals from the same family) was a major clinical observation.

We have shifted technical aspects of the viral load estimation so to put forward these two points regarding the false-negative estimation.

[...] with modeling taking into account an estimate of actual prevalence at the time of testing.

We have added a full discussion regarding the evolution of the viral load within individuals in Sec. Sec. I.2.4. Interpretation of the Gaussian mixture model, page 9. This formalism allows for a prediction of the actual prevalence at the time of result collection.

1. The framing of the article is odd and fails to account for understanding of infectious disease epidemiology. Although it is true that pooled testing could be used to estimate the prevalence of COVID-19, prevalence of an infectious disease with such high transmission potential is an odd metric to use in surveillance. As soon as the prevalence is estimated, it would be outdated. I recommend reframing the article around a realistic use of pooled testing, either to increase throughput of incident cases or as screening in attempts to find SARS-CoV-2 infections and break chains of transmission.

Following the reviewer 2 suggestion, we have reframed our introduction to orient the question of the interest of pool testing to the context of regular community surveillance and on large-scale screening campaigns.

Regardless of the interest of prevalence estimation in the peculiar Covid-19 context, we wish to point out that sample pooling is an efficient tool to achieve a survey campaign designed to measure the prevalence at a very high precision with the most limited usage of tests. In this case, the immediate objective of the sample pooling survey is not in breaking chains of transmission - since at the optimal pool size N = Nopt, 80% of pools turn positive, it may seem impractical to associate the result of pool testing to targeted isolation protocols. In this case, we believe that the measured prevalence is at least of a rough estimate of the current measure prevalence.

In parallel to us, a similar objective was considered by a team (including infectious disease epidemiologists) in a recently posted preprint: *Cleary, B. et al. (2020) 'Using viral load and epidemic dynamics to optimize pooled testing in resource constrained settings', medRxiv.*

In resource constrained settings, using tests to measure the prevalence among asymptomatic individuals (rather than for diagnosis of symptomatic individuals) may appear ethically challenging. We do wish to address such issues in our manuscript.

Although we agree that monitoring alone cannot prevent the onset of an epidemic outbreak, regular prevalence estimates could trigger policies (e.g. confinement measures, bars and restaurant closure) indented at breaking chains of transmissions.

We think the proposed protocol in the new Sec. II. 2. Risk mitigation from a cluster of infected individuals page 15 is realistic; such protocol was discussed with a research institution interested in obtaining regular measures of the prevalence.

a. Lines 2-6. Regularly monitoring the prevalence of the disease does not prevent the onset of an epidemic wave. Prevalence means nothing without incidence, and monitoring does nothing to prevent increasing transmission. Also prevalence is not the metric that would be used to assess the effectiveness of interventions.

The reviewer convinced us to thoroughly change our introduction and our motivation.

We agree that monitoring alone cannot prevent the onset of an epidemic outbreak. However, monitoring can help institutions to scale their response within a predefined graded response scheme - e.g. in a college/university campus context, a rising measured prevalence could serve as a metric to decide whether collective sports events may or may not take place, or, at higher levels, whether to switch to remote teaching.

Based on the reviewer suggestion, we now mention that having on-the-fly estimate of the prevalence based on the currently available results of pools is useful on one aspect, that is to readjust the pool sizes to a near optimal level for individual diagnosis.

b. Lines 20-23. Although it is true that repeated random samples would give a measure of the prevalence of the pathogen, prevalence is not used to monitor infectious disease unless the carriage is long (such as tuberculosis, malaria, or HIV). With SARS-CoV-2 having about a two-week infectious interval, prevalence becomes basically worthless.

We believe that estimating the prevalence has at least one practical implication -- the estimation of the optimal pool size needed for diagnostic purposes. The prevalence may be estimated on a daily basis, such that the measured prevalence among a subset of a population at a given date T might be informative of the measured prevalence among another subset of the population at the date $T + 1$ day, especially in a context of a slowly evolving epidemic with doubling times generally in the 10-20 days range.

We agree that breaking chains of transmission is indeed the objective of screening campaigns, but such objective might not be incompatible with measuring a prevalence - some institutions are indeed interested in such measure:

(1) Shortly after our initial arxiv submission, the European Center of Disease Control has issued a notice to measure the prevalence using pool testing. We now briefly discuss the similarity of our approaches in the main text.
(2) In UK, the survey company Ipsos has performed 5 wide-scale testing among 600 000 citizens; the results of the survey were summarized by a prevalence index.
(3) The SUNY (State University of New York) Covid 19 online tracking application tracks the measured prevalence with time (we point out that SUNY massively uses pool testing).

Mass testing instead, should be used as an intervention to screen and find infections. Pooled testing is also useful to increase throughput for diagnostic testing.

We fully agree with the referee's comment and reformulated the introduction accordingly.

c. In section 1 the authors presume a point prevalence of 3%. A point prevalence of 3% for SARS-CoV-2 would be alarmingly high and would signify that the transmission rate is out of control. I would recommend at the highest using 1%, and if really trying to

We understand the reviewer concerns regarding the choice of a 3% prevalence for the former Figure 2 (now changed into Figure 4, following the re-ordering of sections).

We hope our choice of this value was not misinterpreted as a lack of epidemiological understanding - nor as a confusion with positivity rates.

Based on the Reviewer suggestion, we have changed Figure 1 (now Figure 6 page 18) and we set the prevalence at 1% (which is still high, but compatible with the value measured in the general population in Slovakia or Liverpool, for instance).

What decide us to choose a high level for the prevalence as the objective of former Figure 2 was the need to illustrate the optimality of the pool size - a 3% prevalence is large enough for the optimal pool size to lie below 100, i.e. in a technically feasible range (large pool sizes have been investigated in Mutesa et al. Nature 2020). Should we have chosen a lower value of the prevalence, as we now consider in Figure 6, the estimated optimal pool size lies at very large values Nopt > 255.

Unfortunately, we would like to add that a 3% prevalence has been measured among asymptomatic health care personnel (ranging from 1% to 7%, see in particular Treibel et al. 10.1016/S0140-6736(20)31100-4 and references mentioned in Hogan et al.). In addition, a 3% point prevalence may have been reached even within the general population in a few cities in France (Roubaix or Saint-Etienne) within the current second wave. We do agree this is alarmingly high.

We also added within the SI, page 30, several figures corresponding to different values of the prevalence.

2. Another example highlighting the lack of infectious disease epidemiology understanding is in lines 10-13. This metric is called test positivity, and corresponds to the proportion of tests that are positive among treatment-seeking individuals. The term apparent prevalence is not widely used, and should not be continued. Although the principles discussed in this paragraph are correct, the terminology is wrong and confusing. I suggest reframing this paragraph to discuss how test

positivity among treatment seeking individuals will overestimate the prevalence of the pathogen in the population.

We followed the Reviewer suggestion and removed the mention of apparent prevalence.

We fully agree with the referee that the distinction between test positivity and prevalence is particularly critical. By no means do we want to promote confusion between these two concepts. What we try to convey is that the test positivity significantly overestimates the prevalence of the pathogen in the population; at the current time of manuscript completion (November 24th 2020), the positivity rate in medical laboratories in France is around 10%, while the national prevalence is expected to lie below or around 1%.

We have added a larger paragraph in the introduction with a clear distinction between screening & diagnostic test (as defined by the US CDC), page 2.

3. The authors' focus on the application of group testing to solve the problem of estimating the prevalence is strange. The problems of estimating the prevalence of a pathogen are rooted in who gets tested and how to draw the sample rather than in how the test is conducted. Pooled testing increases throughput, which then can operationalize testing to screen for more infections.

We have significantly rephrased our introduction to shift the focus on screening (section surveillance) and removed any broad epidemiological statements regarding the interest of the prevalence measurement.

We thank the referee for her/his useful comments for convincing us to change the focus of our work into the more relevant one of breaking transmission chains.

Reviewer #3: Review of “Group testing as a strategy for COVID-19 epidemiological monitoring and community surveillance “

The paper analyses the use of group or pooled testing for detecting COVID-19 prevalence in a population. It assumes a limited number of available tests, and looks at the optimal number of individuals to pool together into each test to achieve tightest confidence interval around the estimate of prevalence.

This is a solid and interesting paper, and it addresses all the major problems associated with pooled testing.

We thank the reviewer for these positive comments.

I have a few minor comments.

1. Line 90, the paper cites a “classical computation”, giving a 2020 reference. If it is a classical computation, I'd cite an earlier source. The source cited, does not, as far as I can tell, use the same computation. Instead it uses c_{*}=1. Please give a correct reference, and even better an actual derivation of the result.

We thank the reviewer for pointing out this referencing mistake. To the best of our knowledge, the first occurrence of that computation is in:

Thompson, K. H. (1962) ‘Estimation of the Proportion of Vectors in a Natural Population of Insects’, *Biometrics*, 18(4).

which was rightfully cited at the top paragraph, but incorrectly cited near the calculation of Nopt. Following the reviewer’s suggestion, we have added a short derivation of the result with the SI Sec I.A. page 28.

This result seems indeed valid for reducing the confidence interval, though there are other possible choices for error reduction, such as mean square error, or, my personal favorite, maximum information. As the paper points out, the minimum is very flat and as such these are very similar.

Indeed, these methods provide equivalent results asymptotically (large survey sizes). In particular, shortly after our initial arxiv submission, the European Center of Disease Control produced a similar calculation for the prevalence confidence intervals based on the Delta method. We now briefly discuss the asymptotic identity between our results in the main text, in red page 18.

2. The paper derives a distribution of C_{t} values, the viral load. What is not taken into account or mentioned is that this distribution is not a constant. Each individual goes through a time course

of viral load, and therefore the distribution depends on the time course of the disease in the population.

We fully agree with this statement.

We have added a full subsection Sec. I.2.4. Interpretation of the Gaussian mixture model, page 9 within our manuscript regarding the viral load evolution that also provides an interpretation for our analysis of the two-Gaussian distribution.

We believe this point is important to understand why the asymptomatic population exhibits a more spread distribution in viral loads. Such heterogeneity in viral loads, which is well encompassed by our multi-Gaussian decomposition, has important consequences for public health policies -- tests that miss low viral loads are generally assumed to have low performances on asymptomatic individuals.

As a perspective, we think that, building upon our model, it should be possible to calibrate different viral load datasets that would be obtained in different epidemic contexts. However, as we lack epidemiological context on all available datasets, we would prefer to leave such analysis for future work.

Our censored-Gaussian fitting with epidemic stage adjustment now plays a more explicit role in the “large-scale screening” section (previously Sec. III, now Sec II).

I don't see a need to use this fact in the estimation [*of the viral load evolution*], but it would be nice to mention this fact, and maybe address how results would differ, if they would.

We added a critical discussion on this point which further clarifies why two to three Gaussian lobes are observed (see also discussion in the previous point).

In particular, Hay et al. 10.1101/2020.10.08.20204222 provides an evolution of the viral load within a (relatively large) hospital (Brigham Women’s Hospital in Boston) across the epidemic first wave. The population-level viral load was weak during summer (low viral circulation periods). We refer to the graph below which provides an estimated number of infection (red), effective reproductive number Rt (green) and viral load distribution.

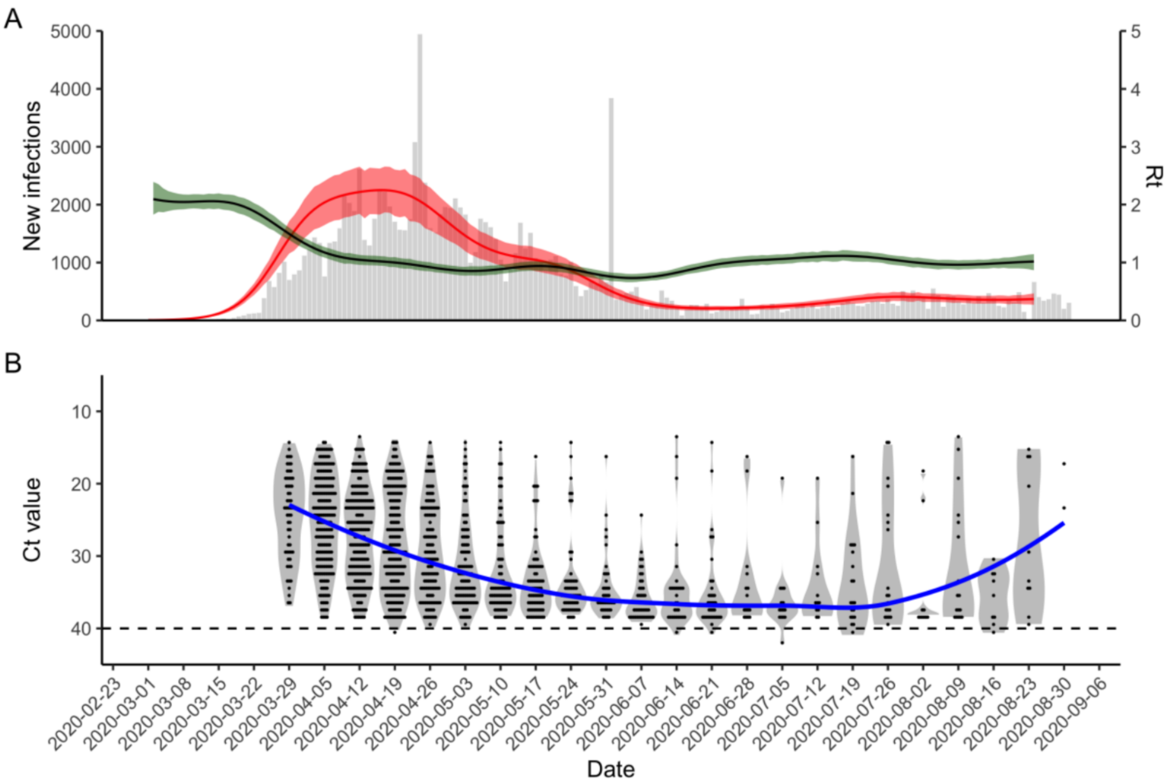

In my opinion all of section 3 detracts from the main message of the paper. I think it should go in a supplement, just giving the main result of mixture of Gaussian + censure in the paper for further analysis.

Following the Reviewer suggestion, we streamlined the section, shifted a significant fraction of the materials to the SI so that the mathematical procedure fits within a single page. We found it difficult to reduce the main text content even further as the naive/censored fitting procedure (1) is relatively tricky to explain and (2) is one of the main mathematical result of the manuscript.

3. Section 4.4.2, optimization of regularity of test, stands somewhat apart of the rest of the paper. It is a very interesting question, but the paper only addresses it via simulation.

Following the reviewer suggestion, we have shifted the former Section 4.4.2 and the corresponding Figure to the SI, page 44.

We have also strengthened the analytical calculation of that section.

There is also no discussion of false positives, which are relevant for timing of outbreak.

The risk of false positives is indeed a major concern in large-scale screenings. With a 1% false positive risk associated with rapid antigen tests, the corresponding positive predictive value is high at low prevalence; this represents a frequent concern regarding the acceptability of positivity-induced-isolation among the population.

However, false positives seem rare with RT-qPCR nasopharyngeal tests; a 99,9% estimate is mentioned in Hogan et al. based on an absence of subsequent development of antibodies.

We expect such rates to be equivalent in pools as long as the threshold viral concentration is not too significantly reduced.

We added a brief discussion regarding this aspect in the main text, page 15: *Here, we focus on the false negative risk. False positives are also a concern in low prevalence settings whereby positive predictive value might be low. However, positives appear very rare in RT-qPCR tests - with an estimated higher bound at 0.01\% \cite{Hogan2021}.*

4. The paper uses “contaminated individual” for “infected individual”. Usually, I would use contaminated for a false positive.

We have corrected the denomination “*contaminated*” into “*infected*”.

There are also some other small problems such as “law of the artefact” on line 138 - I'm not sure what this means, “exemple” on 105.

We removed that term in favor of “false-positive law”. We had used the terminology of a specialized paper on the RT-qPCR technique but we agree that such term was needlessly complicated in the current context.

“But to reach similar level of precision than in single testing“ 107.
“the measure is always made for samples detected as positive “ 240.

We removed these odd sentences.

“We now show how the previous analysis of the tests used to measure the viral load in patients can be used to precise the epidemiological monitoring of the disease in the general population. “ 330.

We reformulated this sentence.

I think these are errors, but since I'm not a native English speaker, I'm not totally sure. Careful editing would be good.

We did an extended proofreading of the manuscript.

We thank the reviewer for his/her helpful comments and positive feedbacks.
