# Supplementary material for: Group testing as a strategy for COVID-19 epidemiological monitoring and community surveillance
Source: PLoS Comput Biol. 2021 Mar 4;17(3):e1008726. doi: 10.1371/journal.pcbi.1008726 (PMC7932094; doi:10.1371/journal.pcbi.1008726)
Supplement: Attachment — Submitted filename: Reviewers_120121.pdf [file pcbi.1008726.s005.pdf]

We are delighted to read the referee comments.

Reviewer #1: As with my initial review, I've limited my focus to the conceptual approach, interpretation, and potential implementation of the work for disease surveillance. I find acceptable and agreeable the author's edits made in response to my suggestions, and furthermore agree with those made in response to the other reviewers as well.

We thank Reviewer 1 for these positive feedbacks.

Reviewer #2: The authors have done commendable work revising the article and responding to my edits. I really enjoyed the introduction, and again incorporating false negatives into the modeling is extremely well done. I am satisfied with their response I include a few more revisions that stood out, all minor.

We thank Reviewer 2 for these positive comments and further suggestions.

1. Lines 28-30 could be revised for clarity. Completely asymptomatic infections only occur at most in 20% of the population, whereas pre-symptomatic transmission is quite common. We don't know yet how much asymptomatic carriers actually transmit coronavirus, but we do know that pre-symptomatic transmission is common. I suggest striking this sentence beginning, "particularly challenging…" and having the previous sentence start the new paragraph with China as the example. There are two systematic reviews for proportion of cases that are truly asymptomatic that would be better references – both found about 15%. One's now a living systematic review at PLOS Medicine: https://www.ncbi.nlm.nih.gov/pmc/articles/PMC7508369/ and the other is at the Journal of the Association of Medical Microbiology and Infectious Disease Canada: https://jammi.utpjournals.press/doi/10.3138/jammi-2020-0030

We have adjusted this paragraph following the Referee 2 suggestion. Indeed, the previous formulation lacked clarity on the respective importance of (truly) asymptomatic vs. pre-symptomatic individuals in contagion.

Here is how the new paragraph reads:

Line 27

As COVID-19 infected individuals may be contagious without showing symptoms, tracing is particularly challenging; while individuals showing no symptoms throughout the infection appear to account for only 15% of infections~\cite{Byambasuren2020,Johansson2021,Buitrago-Garcia2020}), pre-symptomatic infections appear to cause around 50% of infections, approximatively \cite{Mizumoto2020,Bi2020,Bai2020,Ferretti2020}.

Large-scale testing programs aim at addressing such challenge by allowing an earlier identification of asymptomatic and pre-symptomatic carriers \cite{Lavezzo2020}.} In China, city-wide testing programs were reported in several cities

We have reviewed and added references to the two systematic proposed by Reviewer 1 as well as an additional one, Johansson et al. JAMA Network Open (10.1001/jamanetworkopen.2020.35057).

2. Line 38 should read "seminal" rather than "semina"
3. Line 87 – This local newspaper article is probably the best write-up of how SUNY is doing their massive testing and could be cited to give some credit to Dr. Middleton's heroic efforts: https://www.syracuse.com/coronavirus/2020/11/how-upstate-medical-university-used-spit-and-grit-to-make-game-changer-coronavirus-test.html

Thanks for sharing this article, we had missed. We now cite it as a reference for SUNY testing campaign.

Scientists at the University of Chicago also encountered similar challenges: https://www.captodayonline.com/making-peace-with-saliva-pooled-testing/
